# Supplementary material for: Deficiency for scavenger receptors Stabilin‐1 and Stabilin‐2 leads to age‐dependent renal and hepatic depositions of fasciclin domain proteins TGFBI and Periostin in mice
Source: Aging Cell. 2023 Jun 25;22(9):e13914. doi: 10.1111/acel.13914 (PMC10497815; doi:10.1111/acel.13914)
Supplement: Supplementary file 1 — Figures S1–S4 [file ACEL-22-e13914-s002.zip › Aging_cell_R1_Supp_no_markup.docx]

**S. Fig. 1** Survival curves are similar for both male and female mice

**(A)** Survival curve of male (black, left, n=10) and female (red, left, n=30) Stab-DKO mice and Stab-POSTN-Triple deficient mice (right, n=9 for male and female mice).

ns = not significant, *= p<0.05; **=p<0.01; ***=p<0.001.

**S. Fig. 2** Specificity of POSTN antibody in kidney and liver

**(A)** Representative photomicrographs of kidney tissue (left panel) and liver tissue (right panel) of Stab-DKO (upper panel) and Stab-POSTN-Triple deficient mice (lower panel) stained with POSTN. **(B)** Representative photomicrographs of Sirius-red stained liver tissue. Quantification of average Sirius-red positive area of total photomicrograph area is shown on the right (in % of total photomicrograph area). n≥5 for all experiments. Scale bar = 50 µm. ns = not significant, *= p<0.05; **=p<0.01; ***=p<0.001.

**S. Fig. 3** Localization of POSTN and TGFBI in liver and kidney tissue

**(A)** Representative photomicrographs of glomeruli of Stab-DKO animals stained with TGFBI (blue) and POSTN (yellow). Scale bar = 50 µm. **(B)** Representative photomicrographs of l glomeruli stained with EMCN (blue), COL1A1 (green) and POSTN (red). Scale bar = 50 µm. **(C)** Representative photomicrographs of liver tissue stained with pericentral LSEC marker EMCN (blue), TGFBI (green) and POSTN (red). Scale bar = 100 µm. **(D)** Representative photomicrographs of liver tissue stained with pericentral LSEC marker EMCN (blue), COL1A1 (green) and POSTN (red). Scale bar = 20 µm.

n≥5 for all experiments. CV = Central Vein, PF = Portal Field, Si = Sinusoids

**S. Fig. 4** Simple Western™ and Western blots of kidney and liver tissue from Stabilin-deficient animals

**(A)** Simple Western™ based quantification of TGFBI intensity relative to total protein content in homogenized liver (upper panel) and kidney (lower panel) tissue. N=6 for all experiments. **(B)** Representative photomicrographs of Western-Blot analysis of TGFBI (upper panel) in homogenized kindey and liver tissue. GAPDH was used as a loading control (lower panel), recombinant TGFBI as a positive control (left lane). Quantification of TGFBI intensity relative to GAPDH intensity in homogenized kidney tissue (middle panel) and homogenized liver tissue (right panel) relative to WT. n≥3 for all experiments. ns = not significant, *= p<0.05; **=p<0.01; ***=p<0.001.
