# Supplementary material for: Deficiency for scavenger receptors Stabilin‐1 and Stabilin‐2 leads to age‐dependent renal and hepatic depositions of fasciclin domain proteins TGFBI and Periostin in mice
Source: Aging Cell. 2023 Jun 25;22(9):e13914. doi: 10.1111/acel.13914 (PMC10497815; doi:10.1111/acel.13914)
Supplement: Supplementary file 1 — Figures S1–S4 [file ACEL-22-e13914-s002.zip › Supplementary_Figures_merged.pdf]

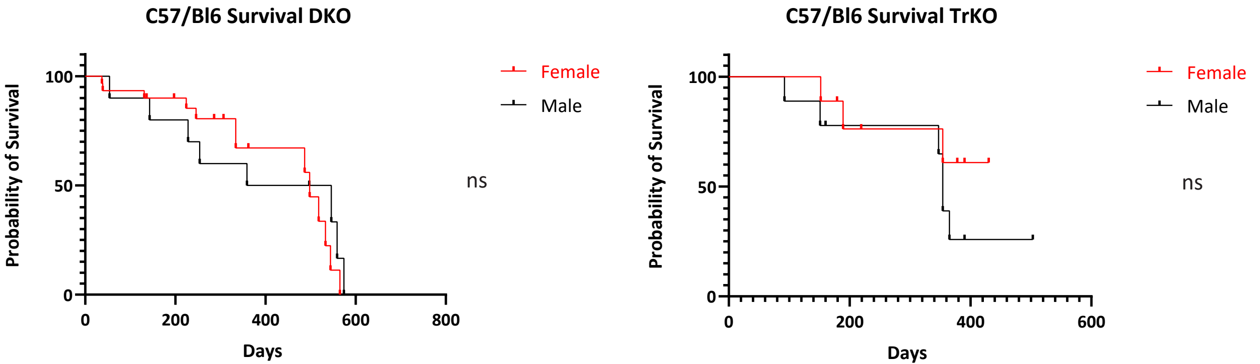
C57/Bl6 Survival DKO
Probability of Survival
100
50
0
0
200
400
600
800
Days
Female
Male
ns
C57/Bl6 Survival TrKO
Probability of Survival
100
50
0
0
200
400
600
Days
Female
Male
ns

Supp. Fig. 1

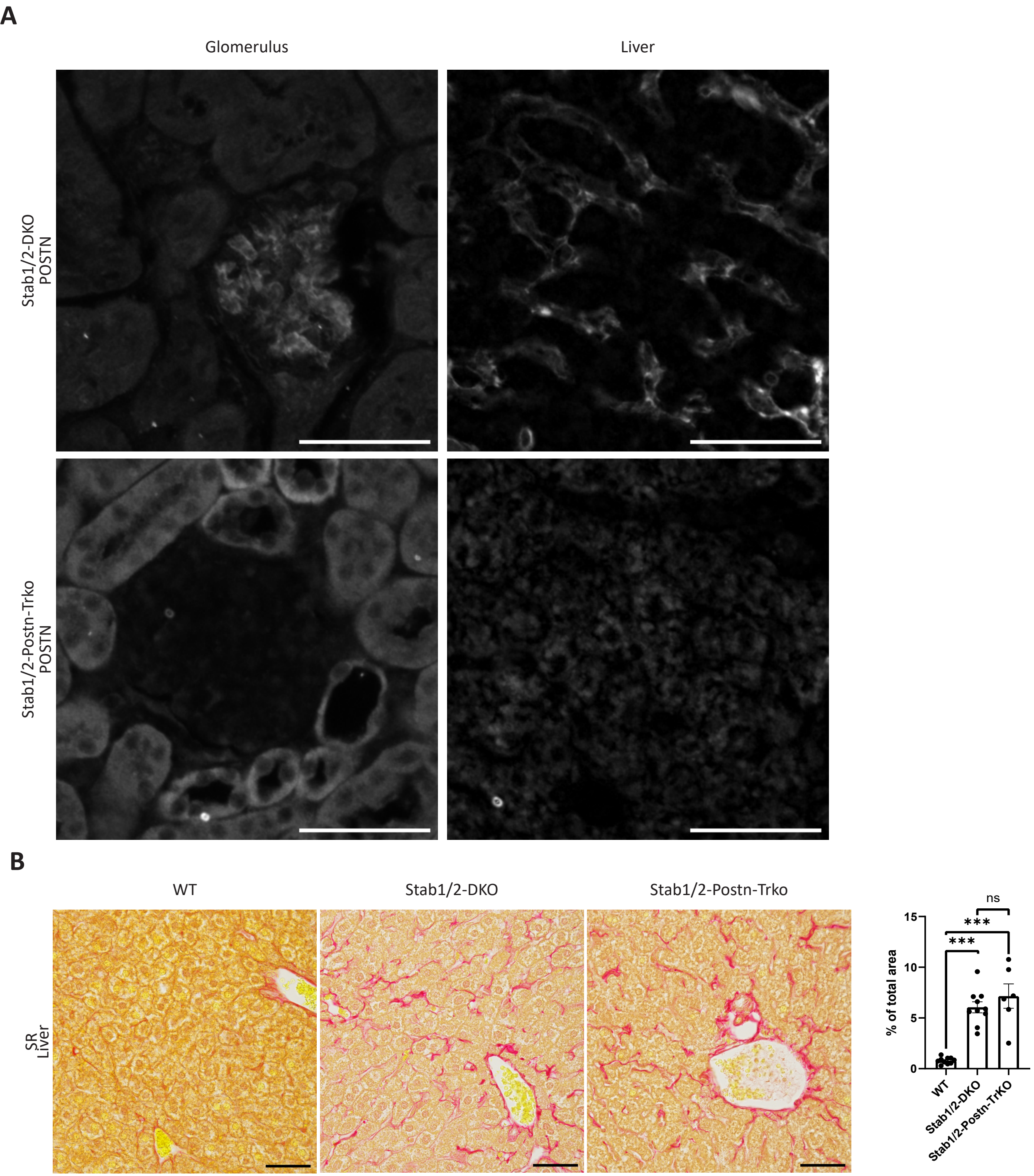
A
Glomerulus
Liver
Stab1/2-DKO
POSTN
Stab1/2-Postn-Trko
POSTN
B
WT
Stab1/2-DKO
Stab1/2-Postn-Trko
SR
Liver
% of total area
15
10
5
0
ns
***
***
WT
Stab1/2-DKO
Stab1/2-Postn-TrKO

Supp. Fig. 2

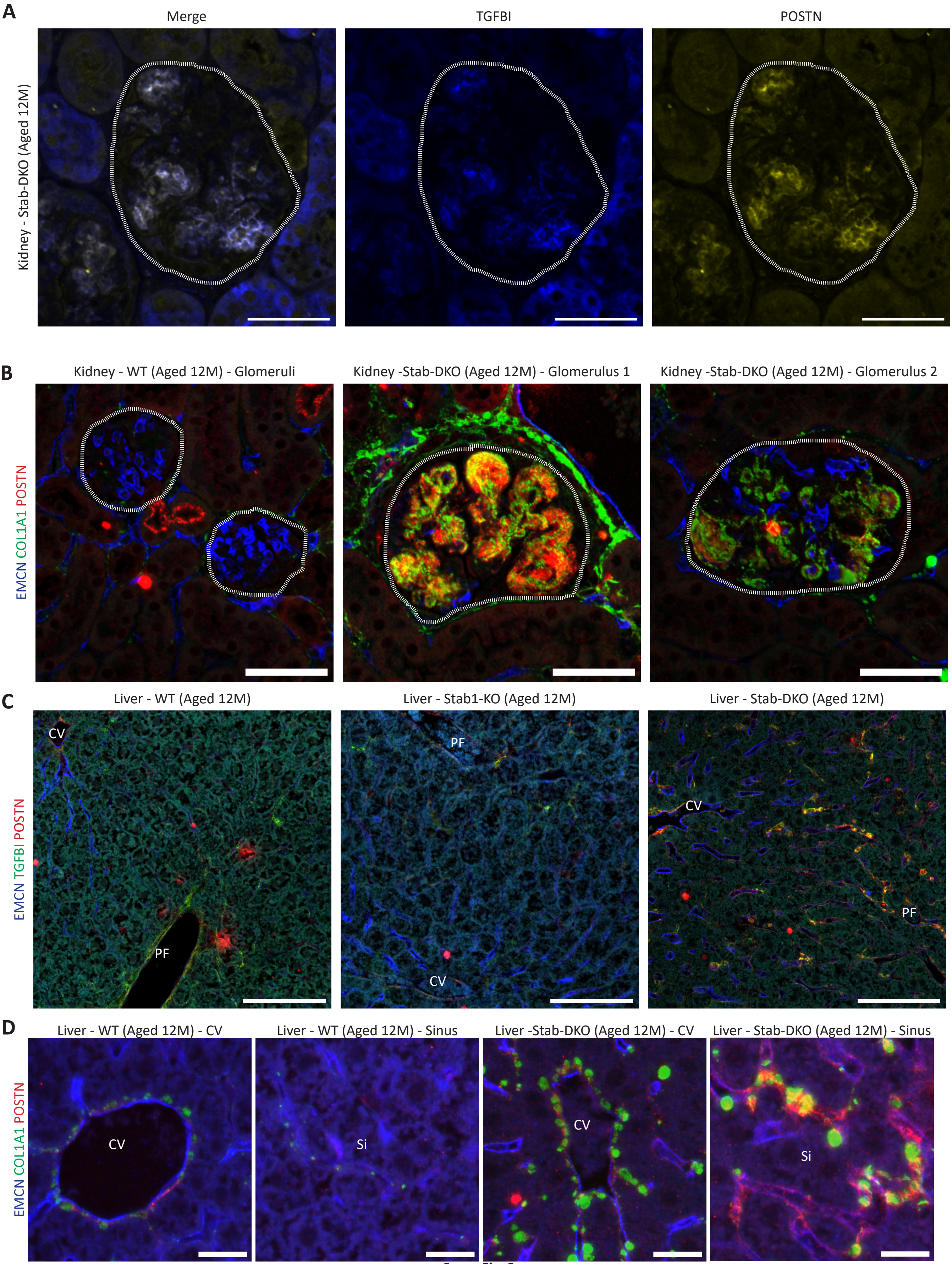
A
Merge
TGFBI
POSTN
Kidney - Stab-DKO (Aged 12M)
B
Kidney - WT (Aged 12M) - Glomeruli
Kidney -Stab-DKO (Aged 12M) - Glomerulus 1
Kidney -Stab-DKO (Aged 12M) - Glomerulus 2
EMCN COL1A1 POSTN
C
Liver - WT (Aged 12M)
Liver - Stab1-KO (Aged 12M)
Liver - Stab-DKO (Aged 12M)
EMCN TGFBI POSTN
CV
PF
PF
CV
CV
PF
D
Liver - WT (Aged 12M) - CV
Liver - WT (Aged 12M) - Sinus
Liver -Stab-DKO (Aged 12M) - CV
Liver - Stab-DKO (Aged 12M) - Sinus
EMCN COL1A1 POSTN
CV
Si
CV
Si

Supp. Fig. 3

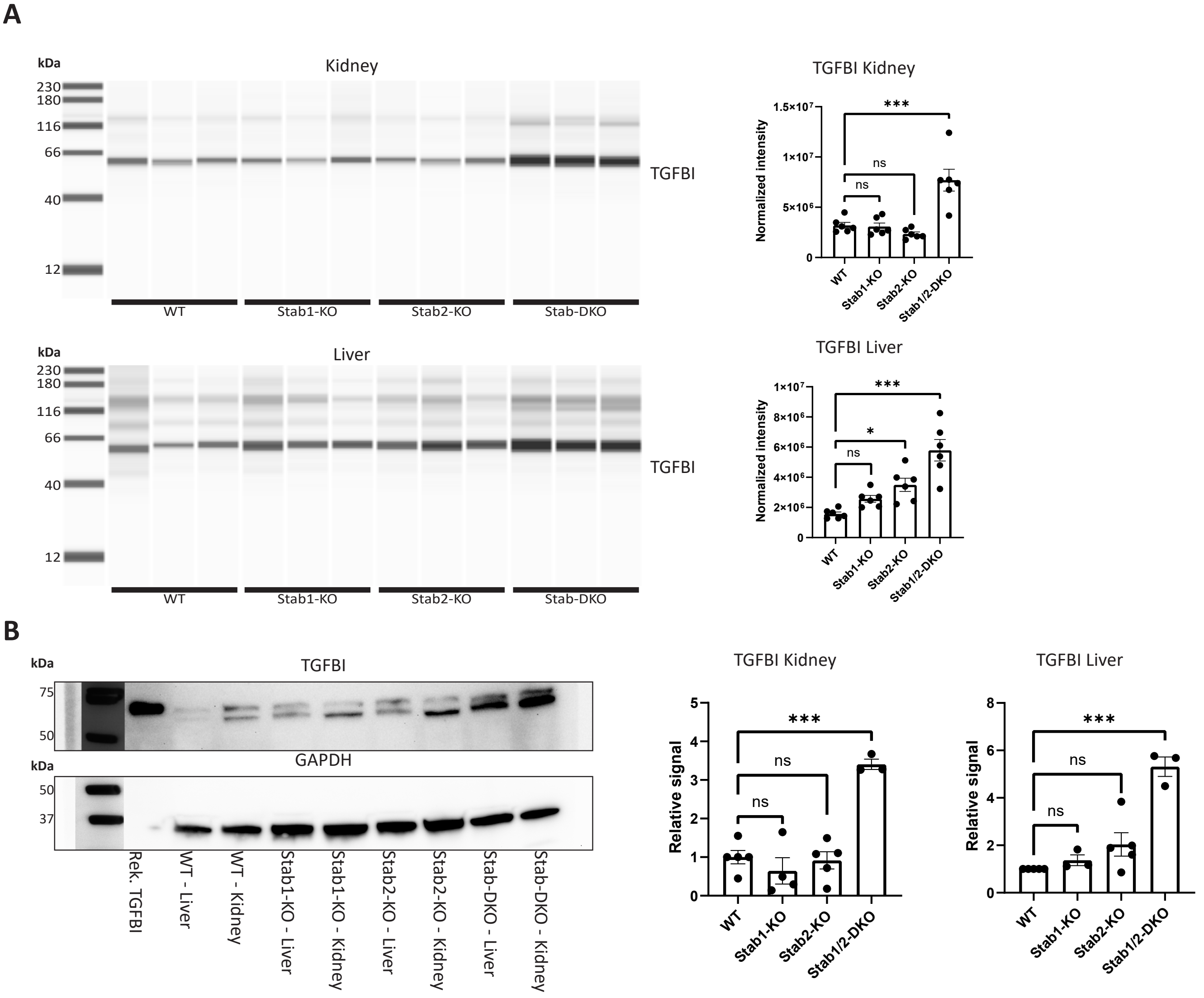
A
kDa
Kidney
230
180
116
66
40
12
TGFBI
WT
Stab1-KO
Stab2-KO
Stab-DKO
TGFBI Kidney
Normalized intensity
***
ns
ns
Stab1/2-DKO
kDa
Liver
TGFBI Liver
*
B
TGFBI
75
50
GAPDH
37
Rek. TGFBI
WT - Liver
WT - Kidney
Stab1-KO - Liver
Stab1-KO - Kidney
Stab2-KO - Liver
Stab2-KO - Kidney
Stab-DKO - Liver
Stab-DKO - Kidney
Relative signal

Supp. Fig. 4
