# Supplementary material for: Deficiency for scavenger receptors Stabilin‐1 and Stabilin‐2 leads to age‐dependent renal and hepatic depositions of fasciclin domain proteins TGFBI and Periostin in mice
Source: Aging Cell. 2023 Jun 25;22(9):e13914. doi: 10.1111/acel.13914 (PMC10497815; doi:10.1111/acel.13914)
Supplement: Supplementary file 2 — Table S1 [file ACEL-22-e13914-s001.zip › ACEL_13914_Supplementary captions.docx]

„Table S1 : qRT-PCR primer pairs, Antibody list, list of RNAScope probes, ImageJ macro used in quantification of glomeruli“
